# Marketing mental health services: a mixed-methods analysis of racially and ethnically diverse college students’ engagement with and perspectives on U.S. university mental health clinics’ websites

**DOI:** 10.1186/s12913-024-11652-2

**Published:** 2024-10-02

**Authors:** Yesenia Aguilar Silvan, Sarah Hamza, Sara Fardeheb, Christine Bird, Lauren C. Ng

**Affiliations:** Department of Psychology, University of California Los Angeles, Los Angeles, CA US

**Keywords:** Marketing, Consumer Engagement, Mental Health Services

## Abstract

**Background:**

The United States (U.S.) faces a significant mental health crisis, with around 52.9 million adults experiencing mental health disorders, with young adults (18–25 years old), such as college students, having the highest prevalence and lowest service utilization rates. While efforts to expand mental health services through “push” strategies are in place (e.g., training therapists in evidence-based therapies), limited initial engagement suggests a need for “pull strategies” and targeted marketing that make services attractive to college students and increase demand. This mixed-methods study identifies U.S. university mental health clinic websites and website characteristics that are attractive and engaging to college students interested in seeking mental health services (i.e., students were considering or actively looking for mental health support).

**Methods:**

Eleven U.S. university websites were chosen (10 randomly and one from the university where students were attending) from a pool of 44 Psychological Clinical Science Accreditation System training clinics websites. Fifty-seven college students (*M*_*age* =_ 20.95, *SD* = 2.97; 81% female; 68% racial/ethnic minority) were videorecorded engaging with two U.S. university mental health clinic websites, completed self-report engagement measures, and gave detailed feedback about websites through semi-structured interviews.

**Results:**

Likert scale scores revealed moderate engagement with all websites (e.g., they were interesting and helpful). Qualitative results indicated that websites that provided important and easily understood information about key features of services (e.g., types, evidence-base, and cost), therapist backgrounds, psychoeducation, used lay language, and had an appealing website layout (e.g., color, font, images, organization, and interactive components) generated greater consumer interest and trust in their mental health services.

**Conclusions:**

This study emphasizes the importance of using marketing strategies to enhance college students’ engagement through mental health service websites. Salient features, psychoeducation, and effective promotional strategies (e.g., how information is presented) were identified as crucial for website engagement and subsequent mental health service uptake. Using marketing strategies, such as tailoring language to consumer literacy levels, describing the evidence-base of services, and improving website design may address college students’ needs and enhance initial mental health service engagement.

**Supplementary Information:**

The online version contains supplementary material available at 10.1186/s12913-024-11652-2.

## Background

Mental health concerns are highly prevalent in the United States (U.S.) [1], with approximately 52.9 million adults having a mental health disorder [2]. Young adults (18–25 years old), including college students, have the highest prevalence of mental health disorders, but the lowest rates of service utilization. To address this disparity, implementation efforts have prioritized “push” strategies to increase access to, and availability of, effective mental health treatments [3]. These “push” efforts include targeting and training therapists and non-mental health specialists (e.g., community health workers) to effectively deliver mental health treatments in diverse settings, such as nonprofits and community health clinics [4, 5]. While such strategies have increased the supply of mental health services offered in the community, initial engagement in mental health treatment continues to be limited [6].

A possible barrier to initial engagement is the absence of “pull strategies” (i.e., consumer-focused promotional strategies) among mental health professionals [7], which could effectively attract people with unmet mental health needs [7–9]. As early as 1988, the American Psychological Association (APA) Division of Psychologists in Public Services called for mental health professionals to market their services to “pull” in clients and raise public awareness about the benefits, safety, and efficacy of psychological services [10]. This strategy remains underutilized, prompting mental health institutions to continue to advocate for marketing to raise awareness about service availability [11]. Historically, health services have used a “one size fits all” approach to recruit clients and geared implementation of services to the “average” consumer [12], overlooking consumer diversity. This nonmarketing approach often fails to reach everyone who might benefit from services and overlooks the underlying reasons for service non-utilization. A shortage of peer-reviewed research and guidance on how to market mental health services likely stymies progress [13].

Targeted marketing offers numerous advantages for mental health services [12], allowing audience segmentation based on characteristics that influence attraction to services and trust in providers [12, 14]. This approach enables researchers and practitioners to reach consumer segments, such as racially and ethnically diverse college students, with specific mental health needs, understand their preferences and cultural beliefs, and create tailored promotional strategies [9, 12, 15]. For example, framing mental health help-seeking as a “first-time experience” in informational messages increased college students’ help-seeking behavior [16], and using love and hope-related terms encouraged consumer engagement and information sharing [17]. These targeted marketing approaches have successfully reached diverse audience segments globally (e.g., racial and ethnic minority communities, low-income families, and sexual minority individuals; [18–22]). Despite these benefits, the widespread use of the internet for health information [23], and established ethical guidelines by respected mental health organizations [24, 25], many mental health professionals remain wary about using online marketing due to privacy concerns [26, 27] and preconceptions of marketing as manipulative [28]. Compared to other health specialties (e.g., physical health, chronic disease, cancer, and sexual health; [20–22, 29–33]), mental health professionals have been slower to adopt marketing strategies to promote their services and facilitate health behavior changes [13].

Further research is essential to fill gaps in marketing research for mental health services and to address discrepancies between mental health professionals’ and consumers’ perceptions of crucial website content (e.g., clinician’s experience and types of services offered) and visual website quality [13, 34, 35]. This substantial research gap, coupled with low mental health service use among college students, presents an opportunity to identify strategies to improve college student recruitment and engagement on mental health service websites. This study used mixed methods to understand college students’ experiences with the marketing of real-world U.S. university mental health clinic websites, by (1) analyzing college students’ distribution of scores on quantitative consumer engagement measures to identify the most and least engaging websites; and (2) use qualitative analysis to identify website characteristics that facilitated and hindered college students’ engagement.

## Methods

### Participants and procedures

Participants (*N* = 123) were recruited through the University of California, Los Angeles (UCLA) Subject Pool from May 2021 to December 2021. Participants were undergraduates interested in seeking mental health services (i.e., students were considering or actively looking for mental health support), ages 18 and above, who had corrected to normal vision. These inclusion criteria were clearly outlined in the subject pool advertisement and reviewed in the consent form, which also provided mental health resources for low-cost services.

An undergraduate-level research assistant obtained informed consent to participate from all the participants in the study through Zoom. After consenting, participants completed baseline surveys through REDCap, with research assistants available for questions. Post-survey completion, participants opted-in to conduct a follow-up appointment where they looked at two randomly selected real-world mental health websites from a pool of 11 websites. The pool consisted of 10 websites randomly chosen from 44 Psychological Clinical Science Accreditation System (PCSAS) clinical training programs (see List of PCSAS Programs in Additional File 1). The UCLA Psychology Clinic, accredited by PCSAS, was the 11th website included due to a larger study aiming to improve service engagement at UCLA’s clinic. PCSAS training clinic websites were selected because they provide low-cost mental health services and focus on research-driven clinical care, facilitating the study’s potential impact on website improvements and dissemination of results.

Within two weeks of the baseline survey, participants who opted into the follow-up appointment (*N* = 57) were randomized to engage with two websites from the pool of the 11 websites. Participants were asked to view and engage with each website as they normally would (e.g., clicking on links and reading content). Afterward, participants completed self-report engagement measures and shared detailed feedback through a 60-minute semi-structured interview. Participants received course credit for completing the survey (one credit) and follow-up appointment (one credit). There were no significant differences in demographics between participants who completed the follow-up interview and those who did not (see Sample Characteristics in Additional File 2). The study was approved by the UCLA IRB (IRB#20-002082).

### Measures

#### Demographic form

Participants completed a 14-item self-report demographic form during the baseline appointment, which included questions about age, gender, race, ethnicity, language spoken at home, place of birth, use of therapy or counseling, use of medication for mental health, and aspects of their life they hoped to improve.

#### Digital behavior change intervention engagement scale

The digital behavior change interventions (DBCI) scale was used during the follow-up appointment to measure and compare an individual’s engagement with a given website by asking a series of ten questions that corresponded to five domains: interest, attention, enjoyment, amount of use, and depth of use [36]. To address this study’s aims, only the experiential items (i.e., the first eight questions) which measure interest, attention, and enjoyment were used. Responses were measured using a Likert, seven-point scale from one (not at all) to seven (extremely). The mean score for each scale and the total mean score were reported. This scale was previously validated with adults living in the UK and had an α of 0.90 for this study sample, indicating high internal consistency.

#### Overall engagement ratings

During the follow-up appointment, participants provided five overall ratings of the clinics’ websites, a process used in prior research [37]. Participants ranked how engaging, helpful, and trustworthy they found the website content, and based on the content how likely they were to seek services if they were struggling with a mental health problem and recommend the webpage to a family member or friend if they were struggling with mental health problems. The ratings were measured on a Likert, five-point scale from one (not at all) to five (extremely). The raw score for each question and an average score for all five questions were reported.

### Semi-structured interviews

Given the limited research on the use of marketing elements to increase initial engagement in mental health services [38], semi-structured interview guides were developed by the first and last author using an inductive approach to describe this phenomenon [39]. The interview had seven open-ended questions and nine probes (see Semi-structured Interview Questions and Probes in Additional File 3).

### Data analytic plan

#### Mixed methods design

A QUAL + quan mixed methods design was used [40], with quantitative and qualitative data simultaneously collected to explore and generate hypotheses. The quantitative analysis assessed engagement levels across websites, identifying the lowest and highest rated websites. These quantitative findings informed the qualitative analysis, which was the primary research method. The qualitative data aimed to determine if there were specific characteristics unique to highly and lowly ranked websites, and how these website characteristics facilitated or hindered engagement in the lowest and highest-rated websites. By building on the quantitative results, the qualitative analysis provided detailed insights into website characteristics associated with varying levels of consumer engagement.

#### Quantitative analysis

The Statistical Package for Social Sciences (Version 28.00; [41]) was used to summarize sample demographics and examine the distribution of engagement scores across all websites viewed. Analysis of variance and post-hoc tests were conducted to identify the clinic websites that had the highest and lowest engagement scores.

#### Qualitative analysis

Data saturation, achieved before 57 interviews, met guidelines [42] with each website viewed by at least six participants (*M* = 10, *SD* = 2.25, range: 7–15; refer to Table 1) and no new codes emerging after coding the first 33 interviews. Using inductive analysis [39], a codebook consisting of a label, definition, and exemplars was developed based on the data. Initial codes capturing emerging key concepts (e.g., website characteristics that impacted engagement) were identified from five interviews, categorized based on how codes were related, and refined iteratively in coding meetings. Definitions were developed for each code and category, and categories were grouped into themes, yielding a final codebook.

**Table 1:** PCSAS websites viewed by participants

Website	Number of consumers
A	8
B	11
C	11
D	12
E	11
F	11
G	8
H	7
I	9
J	15
K	11

Websites viewed between May 2021 and September 2021

A clinical psychology doctoral student (first author) trained two undergraduate research assistants to use the final codebook to code the interviews with the Behavior Observation Research Interactive Software, an open-source software for video coding that allows users to code behaviors (e.g., codes) with timestamps [43]. Due to issues with video configuration, three interviews could not be coded, resulting in a total sample of 54 interviews for the qualitative coding. Research assistants coded five interviews as part of their training and achieved interrater reliability [44] on the fifth interview (Cohen’s Kappa = 0.84), indicating extremely high agreement. Once this level of reliability was reached, they were considered trained and began coding independently. Drift was addressed through regular reliability checks of every eighth interview, during which discrepancies were identified and resolved through consensus meetings. During these meetings, the coding team—comprised of racially and ethnically diverse (Middle Eastern and Mexican) cisgender females with an education in Psychology—leveraged their diverse backgrounds to discuss and reconcile differences in code interpretation. After a one-month break related to university closure, coders rated two booster interviews before resuming independent coding. Coders achieved substantial agreement (Cohen’s Kappa = 0.73) across the five booster interviews. The first author iteratively updated the codebook to include clarifications, a process used in prior research [45, 46] which allows for the refinement of code definitions and collaborative development of themes [44]. For additional details on the qualitative approach, refer to the consolidated criteria for reporting qualitative studies (Additional File 4).

## Results

### Sample characteristics

Participants that completed the follow-up interview (*N* = 57) were approximately 21 years old (*M* = 20.95, *SD* = 2.97), and most self-identified as female (*n* = 46, 81%) and racial/ethnic minorities (*n* = 39; 68%). They were primarily Asian (*n* = 23; 40%), Latine (*n* = 7; 12%), Multiracial (*n* = 7; 12%), and Black/African American (*n* = 2; 4%). Furthermore, a substantial proportion were born outside the U.S. (*n* = 15; 26%) and spoke non-English languages at home (*n* = 25; 44%). Approximately half of the participants had received therapy or counseling (*n* = 32, 56%) and a minority had used medication for a mental health condition in the past (*n* = 11, 19%). Most participants reported wanting to see improvements in their mental health (e.g., managing worries and stress; *n* = 45; 79%).

### Aim 1: Distribution of consumer engagement scores and identifying highest and lowest rated clinic websites

The average DBCI total score (possible range: 1 to 7) was 4.31 (*SD* = 1.25, range: 3.14–5.31), indicating websites were moderately engaging. A Shapiro-Wilk test demonstrated that DBCI scores were normally distributed (*W* = 0.98, *p* = 0.09). The average overall engagement score (possible range: 1 to 5) was 3.12 (*SD* = 1.05: range: 2.13–4.80), indicating websites were moderately engaging. A Shapiro-Wilk test demonstrated that overall engagement scores were not normally distributed (*W *= 0.96, *p* < 0.01), resulting in bimodal scores (“peaks” at a score of 2 and score of 4). Tables 2 and 3 contain the distribution of engagement scores for all websites.

**Table 2:** DBCI total and domain mean scores per website

Website	DBCI Total Average		DBCI Interest Domain		DBCI Attention Domain		DBCI Enjoyment Domain	
	*M*	*SD*	*M*	*SD*	*M*	*SD*	*M*	*SD*
K	5.31	0.94	5.41	1.09	5.70	1.21	4.85	0.95
A	4.89	1.21	5.13	1.22	5.29	1.43	4.33	1.60
B	4.81	1.44	4.41	1.86	5.30	1.32	4.58	1.47
J	4.69	1.30	4.37	1.64	5.16	1.21	4.44	1.40
D	4.60	0.91	4.46	1.12	5.11	1.08	4.19	1.00
G	4.23	0.95	3.94	1.15	4.46	1.08	4.21	1.17
C	4.19	1.07	4.00	1.20	4.73	1.48	3.79	0.92
I	3.82	1.11	3.44	1.86	4.48	1.17	3.41	1.18
F	3.73	1.22	3.36	1.61	4.58	1.44	3.12	1.19
E	3.45	1.24	3.09	1.45	4.24	1.04	2.91	1.41
H	3.14	0.75	2.29	0.86	4.10	1.42	2.76	0.46
All websites	4.31	1.25	4.05	1.59	4.87	1.29	3.92	1.34

Websites are organized by highest to lowest score on the DBCI total score. Scores for each column range from one to seven, with one equaling “not at all engaging”, four equaling “moderately engaging”, and seven equaling “extremely engaging”

**Table 3:** Overall engagement and engagement domain scores per website

Website	Overall engagement Average		Engagement of content		Helpfulness of content		Trustworthiness of content		Seeking services based on website content		Recommending services based on website content	
	*M*	*SD*	*M*	*SD*	*M*	*SD*	*M*	*SD*	*M*	*SD*	*M*	*SD*
K	3.96	0.80	3.45	1.04	4.18	0.60	4.82	0.41	3.82	1.25	3.55	1.51
A	3.80	0.89	3.75	0.89	3.75	1.17	4.63	0.52	3.50	1.20	3.38	0.97
J	3.48	0.90	3.00	1.36	3.60	1.06	4.20	0.94	3.53	0.92	3.07	1.28
D	3.40	0.69	2.92	0.79	3.58	1.08	4.08	0.67	3.17	1.03	3.25	1.03
B	3.38	1.25	3.18	1.25	3.45	1.29	4.18	0.98	3.18	1.54	2.91	1.58
G	3.15	0.70	2.38	0.92	3.25	0.71	4.25	0.71	2.88	0.99	3.00	1.31
C	3.04	0.99	2.82	0.75	3.36	1.29	4.00	0.89	2.82	1.25	2.18	1.47
F	2.69	0.93	1.91	0.83	2.91	0.70	3.64	1.03	2.45	1.30	2.55	1.37
I	2.51	1.18	2.11	1.27	2.56	1.24	3.33	1.23	2.44	1.33	2.11	1.45
H	2.43	0.94	1.57	0.78	2.57	1.13	3.86	1.23	2.00	1.16	2.14	1.22
E	2.13	0.89	1.73	0.91	2.27	1.10	3.09	1.05	1.91	0.94	1.64	1.03
All websites	3.12	1.05	2.66	1.18	3.26	1.15	4.01	0.99	2.93	1.27	2.73	1.40

Websites are organized by highest to lowest score on the overall engagement score. Scores for each column range from one to five, with one equaling “not at all engaging”, three equaling “moderately engaging”, and five equaling “extremely engaging”

A one-way ANOVA showed a significant difference in DBCI total scores by website viewed [*F*(43.25, 133.98) = 3.33, *p* < 0.01]. Tukey HSD tests showed that website K (*M* = 5.31) had a significantly higher DBCI engagement mean score than website E (*M* = 3.45, *p* < 0.01) and website H (*M* = 3.14, *p* < 0.01), indicating website K was more interesting, attention-grabbing, and enjoyable. There were no other significant differences in DBCI scores across other websites.

A one-way ANOVA showed a significant difference in overall engagement scores by website viewed [*F*(34.8, 90.47) = 3.96, *p* < 0.01]. Tukey HSD tests showed that website K (*M* = 3.96) had a significantly higher overall engagement mean score than website E (*M* = 2.13, *p* < 0.01), website H (*M* = 2.43, *p* < 0.05), and website I (*M* = 2.51, *p* < 0.05), indicating website K was more engaging, helpful, and trustworthy. Website E’s overall engagement mean score (*M* = 2.13) was significantly lower than website A (*M* = 3.80, *p* < 0.01) and website J (*M* = 3.48, *p* < 0.01), indicating websites A and J were more engaging, helpful, and trustworthy. There were no other significant differences in overall engagement scores across other websites.

### Aim 2: Qualitative analysis of website characteristics that promoted and hindered engagement

The quantitative findings demonstrated a significant difference in engagement scores across websites, informing the need to compare website characteristics among the highest (i.e., websites A, B, K, and J) and lowest-rated (i.e., websites E, H, I, and F) websites. This comparison revealed website characteristics that either promoted or hindered college student’s engagement with the websites. Certain website characteristics were only found in the highest-rated (e.g., interactive features and testimonials) and lowest-rated (e.g., disclosed the need for session recordings without rationale) websites. The website characteristics were grouped into four overall themes: salient features of services, psychoeducation, optimizing the “buying” experience, and promotion strategies (see summary of themes, categories, codes, and exemplar quotes in Additional File 5 and Additional File 6).

#### Salient features of services

Participants identified key features about mental health services that should be included in university clinic websites to improve engagement and increase interest in seeking mental health services. Key features included providing comprehensive service information (e.g., therapy modalities, service eligibility, adaptations to COVID-19, and evidence-base) and clarity about provider backgrounds (e.g., specialty, demographics, and supervision requirements). Participants reported that irrelevant (e.g., research lab details) and unclear content (e.g., rationale for session recordings) decreased willingness to seek care.

#### Services offered

Participants identified that detailed information about the types of services offered (e.g., therapy, assessments, and intakes), therapy modalities (e.g., telehealth, individual, couples, group, and family therapy), treatment target (e.g., specific disorders), service eligibility, and COVID-19 mitigation practices (e.g., adaptations to service delivery) positively impacted their engagement with the websites. This information allowed them to make informed decisions about whether the clinic would be able to meet their needs and increased their trust in the clinic. Lastly, information about the use of evidence-based practices (EBPs) or treatments increased participants’ willingness to seek services from the clinic. Only the highest-rated websites discussed the use of EBPs.

Participants found that irrelevant information (e.g., research lab and university-related information), and the need to record sessions without a clear rationale or information about deleting recordings after treatment termination, hindered their engagement and reduced their willingness to seek care. Only the lowest-rated websites reported irrelevant information and disclosed the use of session recordings without providing a rationale.

#### Therapist and provider background

Participants identified that detailed descriptions of providers (e.g., including specialties and research interests), and information on graduate students’ supervision requirements increased their willingness to engage with the clinics’ services. A major issue with both the highest- and lowest-rated mental health clinic websites was the lack of clear information about provider roles (e.g., differentiating the roles of clinic staff, faculty, and graduate students was difficult) and details about provider demographics (e.g., race/ethnicity, language, and credentials) and specializations (e.g., working with specific disorders and populations). Participants reported feeling uncomfortable with receiving services from graduate students without details about their training or specializations, an issue noted only in the lowest-rated websites.

### Psychoeducation

Participants noted that overall, both the highest- and lowest-rated websites provided limited psychoeducation about various mental health disorders. They expressed wanting more comprehensive information on mental health disorders, symptoms, and detailed descriptions of treatments that can improve those mental health concerns. The lack of psychoeducation was viewed as a “lost opportunity” for helping participants identify their mental health concerns and seek care.

#### Resources

Participants discussed that including information about external resources, such as self-help recommendations, additional readings on mental health research and symptoms, and emergency services (e.g., hotlines), increased engagement with the mental health websites. Websites without these linked resources decreased participants’ willingness to engage with the clinic’s services. Notably, the absence of emergency crisis numbers was seen as a “missed opportunity” to intervene during crises. The lowest-rated websites often had broken links or lacked descriptions for linked resources, which “intimidated” and concerned participants due to the unknown content of these resources.

### Optimizing the “buying” experience

#### Website goal

Participants identified that mission statements, diversity and anti-racist statements, an “About Us” section, and patient-centered information on the introductory page increased their engagement. They found that helpful summary statements in search engine results could increase engagement. Participants disliked clinic websites embedded within university websites, as it caused confusion (e.g., strayed away from clinic website), and found research-focused (e.g., discussed research studies being conducted within the clinic) or unclear (e.g., target audience) introductory pages less engaging.

#### Financial costs

Participants found information about financial costs, including price ranges for different services, sliding scale details, eligibility for free or low-cost services, and online payment options, helpful for participants when weighing the costs and benefits of engaging with the clinic’s services. Participants wished websites provided more information about service fees (e.g., being able to calculate the specific amount they would need to pay using the sliding scale) and some believed services were too expensive (e.g., some clinics offered $500 assessments).

#### Clinic logistics

Participants reported that clear information about clinic logistics (e.g., location, hours of service, contact information, and confidentiality procedures) increased their willingness to engage with the clinic. Unclear logistics added barriers to seeking care. For example, a website provided the psychology department’s address instead of the clinic’s, leading participants to anticipate that locating the clinic would be challenging. Participants suggested improving the clarity of logistical information.

#### Steps of service delivery

Participants found it helpful when websites provided clear steps for initiating care and outlined the “flow” or service delivery process. They valued knowing what to expect during and after an intake and what documents to bring to first-session appointments. Participants wanted more clarity on how to make appointments and suggested that multiple options to initiate care (e.g., scheduling online or via phone call) could increase ease of use. Participants also desired more information on treatment termination (e.g., how to terminate treatment and repercussions for early termination) to better understand the potential costs of receiving services and make informed decisions.

### Promotion strategies

Participants emphasized that the website design significantly impacted their engagement, aligning with effective promotion strategies. They preferred websites with vibrant, airy, pastel, or warm colors, which they found welcoming and uplifting, optimizing the visual appeal. Conversely, dark or muted colors and excessive white space were disliked for making the websites appear empty. Clear distinctions between foreground and background were important for readability. Effective use of text and font, including bold, italicized, underlined, and large fonts, along with concise text, enhanced comprehension and engagement. Relevant images, such as maps, people interacting with therapists, and diverse staff photos, improved trust and engagement, serving as powerful visual tools. Organized information with clear titles, visible tabs, headers, bullet points, and easily accessible important details increased engagement. Participants suggested adding navigation and interactive features like hyperlinks, dropdown FAQs, filters, surveys, and search bars to personalize the user experience. Lastly, participants favored concise language over jargon and noted a need for multilingual accessibility to broaden the reach and inclusivity of the websites.

#### Color

Participants stated that websites with “vibrant” (e.g., yellow, blue, green, and red), “airy”, “pastel” (e.g., baby blue), and “warm” (e.g., beige and cream) colors were “welcoming” and “uplifting”, making the mental health information more digestible and engaging. On the contrary, they disliked dark, “muted” colors (e.g., gray and navy) and excessive white space, which made websites “appear empty.” Clear distinction between the foreground and background (e.g., tabs should be a different color from the background, and the text color should change when a cursor hovers over) were also important, with only the lowest-rated websites lacking this distinction.

#### Text and font

Participants noted the text and font (e.g., “emphasizing” features such as bolding, italics, underlining, and larger fonts; and reduced text) impacted their understanding and engagement with the website’s information, as it made it either more or less difficult to read.

#### Images and visuals

Participants found that relevant images of maps, therapy interactions, staff (e.g., demonstrating racial/ethnic diversity of therapists and administrative staff), and logos increased their engagement and trust in the website. Participants emphasized that positive imagery such as “uplifting” (e.g., individuals smiling or “looking happy”) and “serene” images (e.g., meadows and nature) evoked a feeling of warmth and being welcomed. However, both the lowest- and highest-rated websites had poor quality (e.g., pixilated and outdated), unappealing or “distressing” (e.g., clinic building that “looked creepy and scary”, people showing negative affect, and empty waiting rooms), and irrelevant images (e.g., images of the brain), which detracted from the website’s appeal.

#### Order and organization of information

Participants reported that “grouping relevant information” with a clear title, visible tabs, headers, and bullet points, improved the order and organization of the information presented on the website, which increased their engagement. They also valued having important information, such as services offered, appointments, and fees, prominently displayed (e.g., top of clinic website) and suggested using navigation features, such as hyperlinks, dropdown menus, and header bars, to improve information access.

#### Interactive components and features

Participants desired more interactive features, such as filters and surveys to assist with registration (e.g., clients filling out concerns, contact information, and availability) and assessing symptoms, on mental health clinic websites to create a more personalized user experience. Only the highest-rated websites had interactive features such as drop-down “Frequently Asked Questions”, while the lowest-rated websites lacked this element.

#### Language used

Participants stated that websites with concise, “straightforward” language, made the information more engaging and easier to read. They found that both the highest- and lowest-rated websites used inappropriate language for “average consumers,” such as jargon and “highly educated language,” and were inaccessible to non-English speakers, suggesting the use of additional languages.

#### Testimonials

Participants found client reviews, recommendations, and testimonials reassuring and helpful, increasing engagement. Only the highest-rated websites included testimonials.

## Discussion

University mental health clinic websites may have a role in reducing the mental health service gap among college students by employing “pull” or marketing strategies. Our study found that consumer engagement varied across websites, with college students reporting website characteristics mattered and influenced their willingness to sign up for mental health care. These findings are consistent with implementation science frameworks [47] that have identified marketing as a strategy to engage individuals in services, ultimately improving the uptake of evidence-based services in community health settings. Our study found that characteristics that highlighted salient features of services, provided psychoeducation, optimized the “buying” experience, and promoted services, increased engagement with the websites, and subsequently the likelihood of mental health service engagement.

Our study found that highlighting salient features of the services increased college student’s engagement. Particularly, the most engaging websites discussed the validity of services. While consumers value information about evidence-based treatments [48, 49], the term “evidence-based” can be unfamiliar and unappealing [50]. Hence, explanations in lay terms are essential for conveying evidence-based treatments effectively (e.g., “therapy that works”; [50]).

Most university training clinics require client consent for recordings or live observations, which may deter consumers [51]. Our findings indicate that university mental health clinics might alleviate concerns about this service feature by providing explanations for recording necessity. College students also reported the importance of disclosing service providers’ details, including demographics, expertise, and cultural training, to increase their willingness to engage in care. Including therapist information on websites can facilitate suitable client-therapist matches, increasing early engagement in mental health services [52]. Furthermore, addressing training and supervision hours for trainee therapists could alleviate consumer concerns about their competence.

College students desired additional resources on mental health literacy and where to seek help on the clinic websites. This may be especially important for racial and ethnic minority consumers, who often lack knowledge about when and where to seek help [53, 54]. It might be beneficial for university training clinics to provide additional information about seeking mental health support and crisis hotline numbers to bolster suicide prevention [55, 56] and facilitate connections to immediate behavioral health care.

Marketing research highlights that convenience in the “buying” experience is key, with consumer introduction to the service being pivotal [57]. Consistently, unclear clinic logistics (e.g., location, contact information, hours of operation, and confidentiality) hindered website ease, indicating a need to address these barriers on clinic websites. College students also suggested that clinics should have a website that is not embedded within a university’s website, which can increase user-friendliness and trust in the service [34]. Disclosing perceived costs or service barriers enhanced appeal [57], which is valuable for college students, particularly those with financial constraints, and racial and ethnic minority clients with transportation and insurance challenges [58]. Clinics should align advertised “low cost” claims with clinic’s current fees and provide clear logistical information. Lastly, mental health intake appointments are common [59] to initiate care, but consumers often find this procedure confusing. Intakes can inadvertently perpetuate lower treatment engagement (Aguilar Silvan Y, Fortuna L, Spencer A, Ng L: Engagement in child psychiatry department appointments: examining the role of social determinant factors and referral pathways, Under Review), thus clear explanations of intake procedures and subsequent care on clinic websites are essential to ease college students’ experience with the website.

Promotion strategies such as communication and messaging, and how information is formatted, sequenced, and reinforced can increase the appeal of services [57]. Despite our sample’s high education level, college students found jargon on the clinic websites making information difficult to understand. In the U.S., approximately 53% of adults struggle with English proficiency, with about 10% being functionally illiterate [60]. To promote low-cost services aimed at community members, clinics must tailor language to the reading level of their target population. All websites were also reported as inaccessible to non-English speakers, perpetuating historical barriers to mental health care [58]. To overcome initial care barriers, clinics should consider offering information in languages relevant to their target population or indicating availability in languages other than English.

Our study also found that website layout, featuring appealing color schemes (e.g., blue, yellow, green, red, and warm and pastel colors) and positive imagery (e.g., photos of staff, people smiling, and nature), increased engagement by creating a welcoming ambiance. Such design choices can target populations of interest (e.g., images of marching soldiers better recruited veterans; [61]) and effectively reach racial and ethnic minority consumers by offsetting stigma-related barriers to care [62]. College students also highlighted the significance of organized information, and navigation and interactive features for increasing website engagement and subsequent service use. Given the consensus that website design, clear layout, and interactive features have a positive effect on trust and credibility [35], university mental health clinics should consider improving their website design to enhance consumer engagement.

College students expressed the value of having testimonials from previous service users to enhance trust in mental health clinics. While for-profit companies and a small percentage of psychologists use this promotion strategy [63], university mental health clinics that have APA accreditation might be less likely to use this strategy because APA has guidelines that discourage clinicians from soliciting testimonials from clients [24]. To foster trust and positive perceptions of treatment, university mental health clinics could emphasize evidence-based treatments in lay terms [64] rather than disclosing client testimonials that may not adhere to the APA guidelines.

### Limitations

Due to the small sample size per website, it was not feasible to conduct further quantitative and qualitative analyses to compare potential differences between consumer demographics. User segmentation is crucial for tailoring recruitment strategies [7, 14, 28]. Future research should examine differences in engagement across various audience segments, including race/ethnicity, immigration status, primary language spoken, and past therapy history, as these diverse user segments within the college student population likely require tailored outreach efforts. These findings might not be generalizable given our sample’s demographics and the selection criteria for clinic websites that were included in the study. Given the sample in our study, it is possible that participants were biased towards rating the clinic website associated with their university as more engaging. Marketing research suggests this would be expected, as participants are more likely to trust information when websites have familiar content features (e.g., logos and color themes) and are created by perceived credible institutions [35].

## Conclusions

In the current study, we examine consumer engagement using mixed-methods data to identify differences across multiple university mental health clinics’ websites. Our study suggests that website characteristics consistent with marketing research were associated with higher consumer engagement on the website and subsequent willingness to engage in the clinic’s services. More research is needed, including qualitative studies of how marketing can be ethically used to increase college student engagement. It is in the best interest of clinic administrators, clinicians, and researchers to listen to college students’ voices and satisfy their needs to increase initial engagement in mental health services. Practitioners and researchers can use marketing frameworks [57] to self-assess the strengths and weakness of an organization or service and prioritize consumer preferences and needs in the design of recruitment and outreach strategies, such as the development of clinic websites. By using these strategies, it is possible that the uptake and implementation of evidence-based mental health treatment can radically improve [47]. Lastly, marketing frameworks are consistent with patient-centered care that encourages policies to emphasize patient views [65]. Patient perspectives can complement healthcare provider perspectives to improve patient experiences and outcomes [65], such as improving the rates of mental health service utilization among racially and ethnically diverse college students.

## Supplementary Information

Supplementary Material 1.

Supplementary Material 2.

Supplementary Material 3.

Supplementary Material 4.

Supplementary Material 5.

Supplementary Material 6.

## Data Availability

The data used and/or analyzed during the current study are available from the corresponding author on reasonable request. Images of the university mental health clinic websites can be provided upon request, contingent on IRB approval.

## Abbreviations

PCSAS: Psychological Clinical Science Accreditation System
DBCI: Digital Behavior Change Interventions
EBPs: Evidence-based Practices
APA: American Psychological Association APA
