# Supplementary material for: Marketing mental health services: a mixed-methods analysis of racially and ethnically diverse college students’ engagement with and perspectives on U.S. university mental health clinics’ websites
Source: BMC Health Serv Res. 2024 Oct 2;24:1163. doi: 10.1186/s12913-024-11652-2 (PMC11446032; doi:10.1186/s12913-024-11652-2)
Supplement: Supplementary file 1 — Supplementary Material 1. [file 12913_2024_11652_MOESM1_ESM.docx]

**List of PCSAS Programs**. On May 3^rd^, 2021, 10 websites were randomly chosen out of 44 programs that were listed on https://www.pcsas.org/accreditation/accredited-programs/ as of May 2021. The UCLA website was added as the 11^th^ website due to the parent study’s research aims. The websites chosen needed to be in-house training clinics associated with the following programs:

1. Arizona State University
2. Boston University
3. Duke University
4. Emory University
5. Harvard University
6. Indiana University of Bloomington
7. McGill University
8. Michigan State University
9. Northwestern University
10. Oklahoma State University
11. Penn State
12. Purdue University
13. Rutgers
14. Stony Brook University
15. Temple University
16. The Ohio State University
17. University of Arizona
18. University at Buffalo
19. University of California Berkeley
20. University of California Los Angeles
21. University of Delaware
22. University of Iowa
23. University of Illinois at Urbana-Champaign
24. University of Georgia
25. University of Kentucky
26. University of Maryland
27. University of Michigan
28. University of Minnesota
29. University of Missouri
30. University of New Mexico
31. University of North Carolina at Chapel Hill
32. University of Oregon
33. University of Pittsburgh
34. University of Pennsylvania
35. University of South Florida
36. University of Southern California
37. University of Texas at Austin
38. University of Virginia
39. University of Washington
40. University of Wisconsin-Madison
41. Vanderbilt University
42. Virginia Tech
43. Washington University in St. Louis
44. Yale University
