# Supplementary material for: Marketing mental health services: a mixed-methods analysis of racially and ethnically diverse college students’ engagement with and perspectives on U.S. university mental health clinics’ websites
Source: BMC Health Serv Res. 2024 Oct 2;24:1163. doi: 10.1186/s12913-024-11652-2 (PMC11446032; doi:10.1186/s12913-024-11652-2)
Supplement: Supplementary file 2 — Supplementary Material 2. [file 12913_2024_11652_MOESM2_ESM.docx]

**Table 1.** *Sample characteristics of study.*

| Variable | Total Sample  (N=123) | | Completed Interview  (N=57) | | Did not Complete Interview  (N=66) | | Statistical Test |
| --- | --- | --- | --- | --- | --- | --- | --- |
|  | *n* | *%* | *n* | *%* | *n* | *%* |  |
| Racial/Ethnic Minorities | 80 | 65 | 39 | 68 | 41 | 62 | χ2(1) = 0.53, *p* = 0.57 |
| Asian | 46 | 37 | 23 | 40 | 23 | 35 |  |
| Latine | 15 | 12 | 7 | 12 | 8 | 12 |  |
| Mixed-race | 15 | 12 | 7 | 12 | 8 | 12 |  |
| Black/African American | 4 | 3 | 2 | 4 | 2 | 3 |  |
| Born Outside of US | 32 | 26 | 15 | 26 | 17 | 26 | χ2(1) = 0.01, *p* = 1.00 |
| Non-English language spoken at home | 55 | 45 | 25 | 44 | 30 | 45 | χ2(1) = 0.03, *p* = 1.00 |
| Self-Identified Female | 104 | 85 | 46 | 81 | 58 | 88 | χ2(2) = 1.96, *p* = 0.34 |
| Had Therapy or Counseling | 65 | 53 | 32 | 56 | 33 | 50 | χ2(1) = 3.07, *p* = 0.10 |
| Used Medication for Mental Health | 33 | 27 | 11 | 19 | 22 | 33 | χ2(1) = 4.63, *p* = 0.59 |
| Wants improvement in Mental Health | 99 | 81 | 45 | 79 | 54 | 82 | χ2(1) = 0.16, *p* = 0.82 |
|  | *M* | *SD* | *M* | *SD* | *M* | *SD* |  |
| Age | 20.95 | 3.39 | 20.95 | 2.97 | 20.95 | 3.74 | *t*(121)=0.01, p = 0.99 |

Note: There were no significant differences between participants that completed the interview and those that did not.
