# Supplementary material for: Marketing mental health services: a mixed-methods analysis of racially and ethnically diverse college students’ engagement with and perspectives on U.S. university mental health clinics’ websites
Source: BMC Health Serv Res. 2024 Oct 2;24:1163. doi: 10.1186/s12913-024-11652-2 (PMC11446032; doi:10.1186/s12913-024-11652-2)
Supplement: Supplementary file 3 — Supplementary Material 3. [file 12913_2024_11652_MOESM3_ESM.docx]

*Semi-structured interview questions and probes.*

|  | Primary Questions | Probes |
| --- | --- | --- |
| 1. | What are your initial thoughts about the website? |  |
| 2. | What do you like about the website? | Why do you like the website? |
| 3. | What don’t you like about the website? | Why don’t you like the website? |
| 4. | What aspects of the website did you find helpful/useful? | What aspects of the website were unhelpful/useful? |
| 5. | How would you improve this website? | Would you change any specific colors?  Would you change any type of visuals?  Would you change the order of information presented?  Would you change the content? |
| 6.  7. | Would you recommend this website to a friend/loved one who was searching for mental health services?  Would you recommend this website to your parents/caregivers? | Why would you (not) recommend this website to a friend/loved one who was searching for mental health services?  Why would you (not) recommend this website to your parents/caregivers? |
