# Supplementary material for: Marketing mental health services: a mixed-methods analysis of racially and ethnically diverse college students’ engagement with and perspectives on U.S. university mental health clinics’ websites
Source: BMC Health Serv Res. 2024 Oct 2;24:1163. doi: 10.1186/s12913-024-11652-2 (PMC11446032; doi:10.1186/s12913-024-11652-2)
Supplement: Supplementary file 4 — Supplementary Material 4. [file 12913_2024_11652_MOESM4_ESM.docx]

Consolidated criteria for reporting qualitative studies (COREQ): 32-item checklist

| **Items** |  | **Guide questions/description** | **Response** |
| --- | --- | --- | --- |
| ***Domain: Research team and reflexivity*** | | | |
| Personal Characteristics | Interviewer or facilitator | Which author/s conducted the interview or focus group? | The first, second, and third author, who were racially and ethnically diverse with different immigrant backgrounds and primary languages spoken at home, conducted interviews. While interviewers were not matched with participants based on their backgrounds, the diversity among the interviewers facilitated culturally sensitive interviewing of diverse college students. |
|  | Credentials | What were the researcher's credentials? | The first and fourth author have a MA degree, the second and third author have a BA degree, and the fifth author has a PHD degree. |
|  | Occupation | What was their occupation at the time of the study? | The first and fourth author are doctoral students in Clinical Psychology, the second and third authors are undergraduate research assistants majoring in Psychology, and the fifth author is an Assistant Professor in Clinical Psychology. |
|  | Gender | Was the researcher male or female? | All researchers are female. |
|  | Experience and training | What experience or training did the researcher have? | All researchers have been trained in qualitative methods, with experience ranging from 2 to 11 years. As part of their training, coders were specifically trained to address biases. During qualitative coding, SH and SF were trained to engage in ongoing discussions to anticipate and interpret biases related to gender, cultural background, and their prior knowledge of marketing mental health services. They learned to address these potential biases as they emerged and to interpret details within the context of cultural meanings and values. |
| Relationship with participants | Relationship established | Was a relationship established prior to study commencement? | No |
|  | Participant knowledge of the interviewer | What did the participants know about the researcher? | Participants knew YA, SH, and SF’s name, credentials, and reasons for doing research. |
|  | Interviewer characteristics | What characteristics were reported about the interviewer/facilitator? | Personal characteristics beyond the interviewer's name, credentials, and reasons for conducting the research were not explicitly disclosed to the participants. However, since interviews were conducted via Zoom, participants had the opportunity to see the interviewers and could make judgments about the interviewer’s gender, race/ethnicity, and cultural background. |
| ***Domain: Study design*** | | | |
| Theoretical framework | Methodological orientation and Theory | What methodological orientation was stated to underpin the study? | Inductive (bottom-up) analysis was used (Hsieh & Shannon, 2005). Building on this methodological orientation, researchers YA and LN, who were sensitive to cultural factors influencing help-seeking behaviors for mental health care services, played a pivotal role in the initial generation of codes during the inductive analysis (consistent with Haitana et al., 2020). Additionally, YA facilitated regular team discussions and reviews to ensure the thorough cross-checking and validation of codes applied by coders. These team reviews included consistent checks for biases related to coders' gender, cultural background, and previous knowledge of marketing strategies. |
| Participant selection | Sampling | How were participants selected? | Participants were recruited through the University of California, Los Angeles Psychology Subject Pool, which is consistent with a convenience sampling method. |
|  | Method of approach | How were participants approached? e | Participants were initially contacted through the University of California, Los Angeles Psychology Subject Pool messaging system. |
|  | Sample size | How many participants were in the study? | 57 participants completed the interview. |
|  | Non-participation | How many people refused to participate or dropped out? Reasons? | 66 participants did not complete the interview because they were not interested in participating. |
| Setting | Setting of data collection | Where was the data collected? | Data were collected through HIPAA-compliant Zoom. |
|  | Presence of non-participants | Was anyone else present besides the participants and researchers? | No |
|  | Description of sample | What are the important characteristics of the sample? | Relevant sample characteristics are presented in Table 1 (e.g., age, race/ethnicity, and past mental health treatment use). |
| Data collection | Interview guide | Were questions, prompts, guides provided by the authors? Was it pilot tested? | Interview questions and prompts are provided in the supplemental materials. The interview questions and prompts were pilot tested among research team. |
|  | Repeat interviews | Were repeat interviews carried out? | No |
|  | Audio/visual recording | Did the research use audio or visual recording to collect the data? | Zoom recordings included audio and visuals. |
|  | Field notes | Were field notes made during and/or after the interview or focus group? | Yes, interviewers made field notes after the interview documenting any potential issues with video and visual quality, or technical difficulties. |
|  | Duration | What was the duration of the interviews or focus group? | Interviews were scheduled to last 60 minutes. |
|  | Data saturation | Was data saturation discussed? | Yes, data saturation (which occurred before 57 interviews) was discussed in-text in the data analytic plan. |
|  | Transcripts returned | Were transcripts returned to participants for comment and/or correction? | No |
| ***Domain: Analysis and findings*** | | | |
| Data analysis | Number of data coders | How many data coders coded the data? | Two. SH and SF coded the data. |
|  | Description of the coding tree | Did authors provide a description of the coding tree? | Yes. Description of the development of the codebook was described in-text in the data analytic plan. Table 6 contains code definitions. |
|  | Derivation of themes | Were themes identified in advance or derived from the data? | Themes were derived from the data. |
|  | Software | What software, if applicable, was used to manage the data? | Behavior Observation Research Interactive Software (BORIS) was used to manage qualitative data. |
|  | Participant checking | Did participants provide feedback on the findings? | No |
| Reporting | Quotations presented | Were participant quotations presented to illustrate the themes / findings? Was each quotation identified? | Yes, supplemental materials contain exemplar quotes of codes. Each quote is identified with a participant ID. |
|  | Data and findings consistent | Was there consistency between the data presented and the findings? | Yes |
|  | Clarity of major themes | Were major themes clearly presented in the findings? | Yes, themes are presented in-text in the results section. |
|  | Clarity of minor themes | Is there a description of diverse cases or discussion of minor themes? | Yes, discussion of categories are presented in-text in the results section. |
