# Supplementary material for: Marketing mental health services: a mixed-methods analysis of racially and ethnically diverse college students’ engagement with and perspectives on U.S. university mental health clinics’ websites
Source: BMC Health Serv Res. 2024 Oct 2;24:1163. doi: 10.1186/s12913-024-11652-2 (PMC11446032; doi:10.1186/s12913-024-11652-2)
Supplement: Supplementary file 5 — Supplementary Material 5. [file 12913_2024_11652_MOESM5_ESM.docx]

**Table 5.** *Summary of themes, categories, and codes.*

| **Theme** | **Category** | **Code** | **Definition** | **Subcodes** | **Consumers**  **(n)** | **Appeared in Highest Rated Websites** | **Appeared in Lowest Rated Websites** |
| --- | --- | --- | --- | --- | --- | --- | --- |
| Salient Features of Services | Important Information | Services Offered | Statements related to information or knowledge about different types of services and care, offered by the MH clinic that impacts engagement with the MH website. | *Promoted Engagement* | | | |
|  |  |  |  | Explicitly stated and explained the types of services offered at the clinic (e.g., therapy, assessments, and intakes) | 23 | ✓ | ✓ |
|  |  |  |  | Provided information about therapy modalities (e.g., telehealth, individual, couples, group, and family therapy) | 14 | ✓ | ✓ |
|  |  |  |  | Stated validity of services (e.g., EBPs and backed by research) | 3 | ✓ |  |
|  |  |  |  | Explicitly stated treatment targets (e.g., specific disorders) | 9 | ✓ | ✓ |
|  |  |  |  | Discussed eligibility for services | 3 | ✓ | ✓ |
|  |  |  |  | Addressed COVID-19 concerns (e.g., adaptations to service delivery) | 6 | ✓ | ✓ |
|  |  |  |  | *Hindered Engagement* | | | |
|  |  |  |  | Limited information about the types of services offered | 15 | ✓ | ✓ |
|  |  |  |  | Included non-service-related information | 2 |  | ✓ |
|  |  |  |  | Disclosed the need to record sessions without providing a rationale and information about session recordings after treatment termination | 2 |  | ✓ |
|  |  | Provider Background | Statements related to information or knowledge about therapist's, provider's, and clinician’s background that impacts engagement with the MH website. | *Promoted Engagement* | | | |
|  |  |  |  | Clearly described who were the providers (e.g., differentiated between staff and therapist) | 12 | ✓ | ✓ |
|  |  |  |  | *Hindered Engagement* | | | |
|  |  |  |  | Unclear or no information about who were the providers (e.g., clinic staff, grad students, faculty) | 9 | ✓ | ✓ |
|  |  |  |  | When providers were identified, descriptions lacked details about provider demographics (e.g., race/ethnicity, language, credentials) and specialization (e.g., target disorders) | 6 | ✓ |  |
|  |  |  |  | Disclosing that grad students will be providing services without provided details about grad student training/specialties | 4 |  | ✓ |
| Psychoeducation | Resources |  | Statements related to information about resources that impacts engagement with the MH website. These include resources that are provided outside of the standard MH clinic services. | *Promoted Engagement* | | | |
|  |  |  |  | Provided some linked resources (e.g., self-help, research, information about symptoms) | 10 | ✓ | ✓ |
|  |  |  |  | *Hindered Engagement* | | | |
|  |  |  |  | Need for additional resources | 10 | ✓ | ✓ |
|  |  |  |  | Provided broken or unhelpful links to resources | 2 |  | ✓ |
|  | Important Information |  |  | Provided little to none psychoeducation about various MH disorders and symptoms | 15 | ✓ | ✓ |
| Optimizing “Buying” Experience | Important Information | Financial Costs | Statements related to information/knowledge about finances that impacts engagement with the MH website. | *Promoted Engagement* | | | |
|  |  |  |  | Provided information about low-cost services (e.g., price ranges for different services, sliding scale, and eligibility for low-cost services) | 18 | ✓ | ✓ |
|  |  |  |  | Provided information about how to make online payments | 4 | ✓ | ✓ |
|  |  |  |  | *Hindered Engagement* | | | |
|  |  |  |  | Fees are expensive | 5 | ✓ | ✓ |
|  |  |  |  | Unclear or limited information about service fees | 13 | ✓ | ✓ |
|  |  | Clinic Logistics | Statements related to information or knowledge about the logistics of the MH clinic that that impacts engagement with the MH website. | *Promoted Engagement* | | | |
|  |  |  |  | Contact information (e.g., provided phone numbers) | 6 | ✓ | ✓ |
|  |  |  |  | Physical location of clinic (e.g., provided directions to clinic) | 16 | ✓ | ✓ |
|  |  |  |  | Hours of service | 9 | ✓ | ✓ |
|  |  |  |  | Confidentiality related forms and information | 5 | ✓ | ✓ |
|  |  |  |  | *Hindered Engagement* | | | |
|  |  |  |  | Unclear or unhelpful information about clinic logistics (e.g., physical location, contact information, hours of operation, and confidentiality) | 18 | ✓ | ✓ |
|  |  | Steps of Service Delivery | Statements related to information about the steps or flow of seeking MH services that impacts engagement with the MH website. This includes statements about the steps needed to make appointments to start services. | *Promoted Engagement* | | | |
|  |  |  |  | Provided information about the first step to initiate care (e.g., making a phone call to make an appointment) | 15 | ✓ | ✓ |
|  |  |  |  | Briefly outlined treatment process for therapy (e.g., steps after intake) | 8 | ✓ | ✓ |
|  |  |  |  | *Hindered Engagement* | | | |
|  |  |  |  | Limited options to initiate care (e.g., only option is to make a phone call) | 7 | ✓ | ✓ |
|  |  |  |  | Unclear or confusing steps to initiate care (e.g., differing steps for different services) | 14 | ✓ | ✓ |
|  | Website Goal |  | Statements related to the goal or purpose of the website that impacts engagement with the MH website. | *Promoted Engagement* | | | |
|  |  |  |  | Engaging introductory page (e.g., diversity statement, patient centered, introduced services) | 20 | ✓ | ✓ |
|  |  |  |  | *Hindered Engagement* | | | |
|  |  |  |  | Website was embedded in university website | 20 | ✓ | ✓ |
|  |  |  |  | Confusing introductory pages | 14 | ✓ | ✓ |
|  |  |  |  | Research-focused introductory pages | 6 | ✓ | ✓ |
| Promotion Strategies | Website Layout | Color | Statements related to the color schemes used on the website that impacts engagement with the MH website. | *Promoted Engagement* | | | |
|  |  |  |  | Appealing color scheme in some website features (e.g., blue, yellow, green, red, and warm and pastel colors) | 13 | ✓ | ✓ |
|  |  |  |  | *Hindered Engagement* | | | |
|  |  |  |  | Unpleasant color scheme used throughout website (e.g., gray, black, dark blue, muted colors | 17 | ✓ | ✓ |
|  |  |  |  | Color of text blended with background | 4 |  | ✓ |
|  |  |  |  | Large amount of white space | 3 | ✓ | ✓ |
|  |  | Text and Font | Statements related to how the text and font of website visually impacts engagement with the MH website. | *Promoted Engagement* | | | |
|  |  |  |  | Proper use of features that emphasize information (e.g., bolding, italics, larger font size, and underlining) | 12 | ✓ | ✓ |
|  |  |  |  | Brief statements | 6 | ✓ | ✓ |
|  |  |  |  | *Hindered Engagement* | | | |
|  |  |  |  | Large amounts of text | 17 | ✓ | ✓ |
|  |  |  |  | Font was unappealing (e.g., size too small and type) | 8 | ✓ | ✓ |
|  |  |  |  | Poor use of features that emphasize information (e.g., bolding, italics, underlining, and asterisks) | 12 | ✓ | ✓ |
|  |  | Images and Visuals | Statements related to the images and visuals of the website that impacts engagement with the MH website. | *Promoted Engagement* | | | |
|  |  |  |  | Included some relevant images (e.g., map, individual speaking to therapist, people smiling/looking happy, and logos) | 20 | ✓ | ✓ |
|  |  |  |  | *Hindered Engagement* | | | |
|  |  |  |  | Had unappealing (e.g., outdated and poor quality images) and irrelevant images (e.g., pictures of brain) | 22 | ✓ | ✓ |
|  |  |  |  | Lacked images (e.g., photos of staff) | 22 | ✓ | ✓ |
|  |  | Organization | Statements related to the order, organization, and structure of information that impacts engagement with the MH website. | *Promoted Engagement* | | | |
|  |  |  |  | Attempted to group information in sections (e.g., used clear heading titles or bullet points to organize information) | 15 | ✓ | ✓ |
|  |  |  |  | Placement of important information (e.g., fees) is visible | 14 | ✓ | ✓ |
|  |  |  |  | Attempted to ease navigation using tabs and headers | 22 | ✓ | ✓ |
|  |  |  |  | *Hindered Engagement* | | | |
|  |  |  |  | Need for improved or additional navigation features (e.g., hyperlinks, dropdown menus, bars, and tabs/headers) | 29 | ✓ | ✓ |
|  |  |  |  | Information poorly placed and grouped (e.g., related information such as types of services not grouped together) | 32 | ✓ | ✓ |
|  |  | Interactive Components and Features | Statements related to interactive components or features that impacts engagement with the MH website. Interactive components refer to features that allow a two-way flow of information between a computer and a computer-user; responding to a user’s input. | *Promoted Engagement* | | | |
|  |  |  |  | Included FAQ section | 10 | ✓ |  |
|  |  |  |  | Hyperlinked sections | 2 | ✓ | ✓ |
|  |  |  |  | *Hindered Engagement* | | | |
|  |  |  |  | Lacked search bar | 2 |  | ✓ |
|  |  |  |  | Lacked other interactive features (e.g., filters and surveys) | 5 | ✓ | ✓ |
|  | Language |  | Statements related to the language used in the websites that impacts engagement with the MH website. This code includes complicated language that leads users to search general web for clarification. | *Promoted Engagement* | | | |
|  |  |  |  | Concise language | 3 | ✓ | ✓ |
|  |  |  |  | *Hindered Engagement* | | | |
|  |  |  |  | Used jargon | 7 | ✓ | ✓ |
|  |  |  |  | Inaccessible to non-English speakers | 9 | ✓ | ✓ |
|  | Important  Information | Testimonials | Statements related to testimonials included from previous clients, users, and service recipients impact engagement with the MH website. | *Promoted Engagement* | | | |
|  |  |  |  | Included testimonials from previous clients | 4 | ✓ |  |
