# Supplementary material for: Marketing mental health services: a mixed-methods analysis of racially and ethnically diverse college students’ engagement with and perspectives on U.S. university mental health clinics’ websites
Source: BMC Health Serv Res. 2024 Oct 2;24:1163. doi: 10.1186/s12913-024-11652-2 (PMC11446032; doi:10.1186/s12913-024-11652-2)
Supplement: Supplementary file 6 — Supplementary Material 6. [file 12913_2024_11652_MOESM6_ESM.docx]

*Theme and code table with exemplar quotes.*

| **Theme** | **Category** | **Code** | **Definition** | **Subcodes** | **Quotes** | **Consumers**  **(n)** | **Appeared in Highest Rated Websites** | **Appeared in Lowest Rated Websites** |
| --- | --- | --- | --- | --- | --- | --- | --- | --- |
| Salient Features of Services | Important Information | Services Offered | Statements related to information or knowledge about different types of services and care, offered by the MH clinic that impacts engagement with the MH website. |  | *Promoted Engagement* | | | |
|  |  |  |  | Explicitly stated and explained the types of services offered at the clinic (e.g., therapy, assessments, and intakes) | “The treatments were very straight-forward, so people can already have an idea about what to expect from this if they were to continue on.” -Participant 9  “Therapy I think is also really great, therapy and testing available, it’s all the things I probably would want to see if I want this service”- Participant 76 | 23 | ✓ | ✓ |
|  |  |  |  | Provided information about therapy modalities (e.g., telehealth, individual, couples, group, and family therapy) | “It’s good because it talks about how it helps adults too and even couples, so parents can use it and grandparents.” -Participant 68  “They’re saying that they have group therapy which is really nice because not everyone does it. And if they do and they don’t advertise it, then it’s hard to know what is offered.” -Participant 82 | 14 | ✓ | ✓ |
|  |  |  |  | Stated validity of services (e.g., EBPs and backed by research) | “It [website] addresses their target audience first then brings out credibility, but not far after, like credibility definitely needs to be there”- Participant 30  “The thing that really makes me trust it is because it’s from a university but also you see evidence-based treatments.”- Participant 59 | 3 | ✓ |  |
|  |  |  |  | Explicitly stated treatment targets (e.g., specific disorders) | “It’s nice to see things they can help treat, which is nice to just know what they’re looking at and knowing how long they think the treatment will last and that it’s tailored to you. That really makes you feel like you’re important and that they’re going to look after you.” -Participant 13  “This is what I really like - how they actually list the different mental illnesses or disorders that they do work out”- Participant 2 | 9 | ✓ | ✓ |
|  |  |  |  | Discussed eligibility for services | “It does a good job of showing that it’s for all age groups and not just for people at [university]. It talks specifically about kids, adults, elderly, and couples. It makes it very clear what they have and who it’s available to.” -Participant 13  “Based off eligibility it could be free which was good to know”- Participant 79  “I liked that they have the specific qualifications to be a patient there.” -Participant 74 | 3 | ✓ | ✓ |
|  |  |  |  | Addressed COVID-19 concerns (e.g., adaptations to service delivery) | “I like how they give the COVID practice updates. At least, even with COVID, they’re still operating on a remote basis so informing the people who use their website.” – Participant 38  “There’s updates here on Covid-19, that’s helpful”- Participant 25 | 6 | ✓ | ✓ |
|  |  |  |  |  | *Hindered Engagement* | | | |
|  |  |  |  | Limited information about the types of services offered | “When I was first on the generalized anxiety page and then I clicked on treatment process because I thought it was going to treatment process tailored for someone who would come in for generalized anxiety, but it wasn’t it was just a general thing so that’s somewhat less helpful. And that’s the case for all of these other treatments”- Participant 45  “I just don’t really understand what exactly they offer here. It’s not clear.” -Participant 60 | 15 | ✓ | ✓ |
|  |  |  |  | Included non-service-related information | “If they could have under their website depression, anxiety, bulimia, bipolar and stuff, that to me would be much more helpful than ‘child studies lab.’ What does that really have to do with me seeking out mental health” -Participant 62  “It’s for people seeking help, I don’t really like that they have information about the student advocacy group because if you’re looking for mental health services, this isn’t really important to you. The only things that will be important to you if you’re looking for mental health services would be the services, the appointment, maybe the payment, and then the mental health resources. – Participant 74 | 2 |  | ✓ |
|  |  |  |  | Disclosed the need to record sessions without providing a rationale and information about session recordings after treatment termination | “If I was seeking mental health services, I might not really want to be recorded. If I was going there, I’d probably feel like I had a pretty serious issue and I feel like I would not necessarily be very happy that it was video-recorded. I feel like that could possibly make me not want to go to this place.” – Participant 35  “Why are they recorded? That’s weird” – Participant 80 | 2 |  | ✓ |
|  |  | Provider Background | Statements related to information or knowledge about therapist's, provider's, and clinician’s background that impacts engagement with the MH website. | *Promoted Engagement* | | | | |
|  |  |  |  | Clearly described who were the providers (e.g., differentiated between staff and therapist) | “I like how it has the description of the different people here and their fields because it shows the diversity of everyone’s focus”- Participant 4 | 12 | ✓ | ✓ |
|  |  |  |  |  | “I like how this one says all therapists are supervised by licensed psychologists”- Participant 2 |  |  |  |
|  |  |  |  | *Hindered Engagement* | | | | |
|  |  |  |  | Unclear or no information about who were the providers (e.g., clinic staff, grad students, faculty) | “They don’t have an introduction of their therapists. It would be cool to see okay this is one of their therapists, a picture of them, and then on the side focuses or areas of concentration, they can help you with like anxiety, bipolar or whatever they can help you with. It’s just ‘make an appointment’ and that’s the first area that I think people would click, so that would turn you away from the website.” Participant 62  I don’t understand the faculty, staff, grad students thing, I guess I don’t know if those are people who are involved with the psychological services center- Participant 45 | 9 | ✓ | ✓ |
|  |  |  |  | When providers were identified, descriptions lacked details about provider demographics (e.g., race/ethnicity, language, credentials) and specialization (e.g., target disorders) | “I see staff, but is staff their therapists that are going to be helping? Because it says clinic staff, is staff their researchers? I know they’re trying to show who they are but it’s almost like it would be nice if they could be like these are the types of therapists you can look at instead of just ‘clinic staff’” -Participant 62 | 6 | ✓ |  |
|  |  |  |  |  | “They mention broadly what services they might offer but I think different clinicians might have different specialties so I really want to get to know them first before I can make a decision if I want to go to this clinic versus another one”- Participant 5  “It might help to have a bit about them or what they specialize in, instead of throwing just a bunch of names at you.” – Participant 13 |  |  |  |
|  |  |  |  | Disclosing that grad students will be providing services without provided details about grad student training/specialties | “It would be helpful I think for them to bring information about what they know and maybe what they specialize in since this is a training center for graduate students. I want to know what they specialize in specifically”- Participant 65  “Another thing I didn’t like is if I’m seeking mental health treatment, I feel like I would want someone who’s fully had their training and it seemed like this particular site was a training center for clinical psychology doctoral students and also undergraduates doing internships here. So, I feel like this is maybe more of a training ground or mentoring site and it might seem like it might detract from the focus on the patient.”- Participant 39 | 4 |  | ✓ |
| Psychoeducation | Important Information | Resources | Statements related to information about resources that impacts engagement with the MH website. These include resources that are provided outside of the standard MH clinic services. | *Promoted Engagement* | | | | |
|  |  |  |  | Provided some linked resources (e.g., self-help, research, information about symptoms) | “Resources, stuff like that, is super helpful. Just having brochures and being able to give a lot of information is definitely helpful so people can be well-informed about what’s going on with themselves, maybe before they even come in. Or if they just want to figure out what’s going on privately I think it’s just good to have.” – Participant 68  “I think it’s good they have external resources to link out to. Just in case this website doesn’t suit you, they still have other resources you can look at.” – Participant 39 | 10 | ✓ | ✓ |
|  |  |  |  | *Hindered Engagement* | | | | |
|  |  |  |  | Need for additional resources | “I think it would be cool if they also add links because a lot of times they don’t have time for appointments. If someone’s suicidal right now, it’s not like they’re going to wait two weeks, like what about in between those two weeks? – Participant 62  “They don’t have any sort of resource number or immediate care number for anyone in crisis which isn’t beneficial”- Participant 45 | 10 | ✓ | ✓ |
|  |  |  |  |  | “Instead of just here you can ‘make an appointment’, what if something’s serious and I’m extremely depressed or having panic attacks, maybe having follow up mindfulness videos on YouTube” – Participant 62 |  |  |  |
|  |  |  |  | Provided broken or unhelpful links to resources | “I don’t really know what this [list of links] is about, so if I had some kind of information from there, maybe I would know that I should visit it. Because this is not informative, it’s just a bunch of links. It kind of looks very dry.” -Participant 82 | 2 |  | ✓ |
|  |  |  |  | Provided little to none psychoeducation about various MH disorders and symptoms | “What was not helpful was them not really including symptoms or what the said disorders are themselves. You would have to do that research on your own and then come to this website with an answer for what you’re specifically looking for”- Participant 66  “It seems, the way they organized it, that they only provide help with pain even though I think it says that they have more than just the pain program  so I guess that probably wouldn't be very helpful for people who are looking for other treatment. But, there’s no information on other problems the way they put information for pain” -Participant 74  “If I was seeking help and I wasn’t really sure what I was specifically going through, it would be hard to understand which services I want to get. So, they need to provide prior information about different problems.” – Participant 9 | 15 | ✓ | ✓ |
| Optimizing “Buying” Experience |  | Financial Costs | Statements related to information/knowledge about finances that impacts engagement with the MH website. | *Promoted Engagement* | | | | |
|  |  |  |  | Provided information about low-cost services (e.g., price ranges for different services, sliding scale, and eligibility for low-cost services) | “It really emphasizes the low-cost part and that [university] students, faculty, and staff receive 10% off which is nice”- Participant 4  “Clinic fees – they talk about a rough estimate for that I think that’s better than the last one because people would have a basic idea of how much it’s going to be so they can see if they can afford it before getting therapy”- Participant 7 | 18 | ✓ | ✓ |
|  |  |  |  | Provided information about how to make online payments | It addresses everything about mental health services and then most importantly, which a lot of other websites leave out, how to pay your bill”- Participant 25 | 4 | ✓ | ✓ |
|  |  |  |  | *Hindered Engagement* | | | | |
|  |  |  |  | Fees are expensive | “I don’t like how expensive the assessments are which makes it very inaccessible to other people”- Participant 66  “I don’t know if that’s really helpful, the price under the assessment is a lot.” – Participant 17 | 5 | ✓ | ✓ |
|  |  |  |  | Unclear or limited information about service fees | “The section about fees doesn’t give you a solid answer.” -Participant 52  “Fees – needs more information, I don’t know what the standard is especially for someone who’s going for the first time for information they don’t want to call and be on hold and then realize they can’t afford it” – Participant 30 | 13 | ✓ | ✓ |
|  |  | Clinic Logistics | Statements related to information or knowledge about the logistics of the MH clinic that that impacts engagement with the MH website. | *Promoted Engagement* | | | | |
|  |  |  |  | Contact information (e.g., provided phone numbers) | It was really nice that they opened up with the contact information right there and I didn’t have to go hunting for it. That really stood out to me and it seemed really inviting so I did find that super helpful” -Participant 51  “I liked how they had the contact right off the bat just front and center because I think that people can be nervous to reach out for help. So, if you have contact button in front of them, it can maybe prompt them to contact more or make them more likely to contact.” -Participant 27 | 6 | ✓ | ✓ |
|  |  |  |  | Physical location of clinic (e.g., provided directions to clinic) | “I liked this address and directions information that they have right here on the home page”- Participant 42 | 16 | ✓ | ✓ |
|  |  |  |  | Hours of service | “The times that they’re open, that’s good because it’s like oh what fits my availability?”- Participant 80  “It was useful seeing their hours right when you click on it because maybe if you’re not already familiar with the website, it would be a simple click and you already have that information so that’s good” -Participant 61 | 9 | ✓ | ✓ |
|  |  |  |  | Confidentiality related forms and information | Confidentiality I think is great because that’s the thing that I’m concerned with probably the most with services like this”- Participant 79  “I think it’s good they talk about confidentiality, too, when they talk about the groups that they work with.” -Participant 62 | 5 | ✓ | ✓ |
|  |  |  |  | *Hindered Engagement* | | | | |
|  |  |  |  | Unclear or unhelpful information about clinic logistics (e.g., physical location, contact information, hours of operation, and confidentiality) | I was confused about it…they didn’t have the availability, time, location, and everything”-Participant 63  “They don’t even have a zip code in their phone number. Also, the phone number is so hidden, how was I supposed to find that? It’s so easily miss-able.” -Participant 53 | 18 | ✓ | ✓ |
|  |  | Steps of Service Delivery | Statements related to information about the steps or flow of seeking MH services that impacts engagement with the MH website. This includes statements about the steps needed to make appointments to start services. | *Promoted Engagement* | | | | |
|  |  |  |  | Provided information about the first step to initiate care (e.g., making a phone call to make an appointment) | “Even simply putting this the appointment tabs is really huge because after reading all this information there’s probably a lot of people that want to move forward and to have it as the first few or big tabs can really help people because you know if people can’t find it or it’s really difficult to obtain it really discourages people from moving forward with the services”- Participant 56  What I actually found really helpful was the appointment section. They had the number right there and you could just call without looking into more information regarding how to make an appointment: you could just call them. I feel like calling or asking questions to another human is always easier than just searching for information online or using an app.” -Participant 12 | 15 | ✓ | ✓ |
|  |  |  |  | Briefly outlined treatment process for therapy (e.g., steps after intake) | “I like this procedures section because it’s like a step by step of what it would be like and people can see what they’re getting into.” – Participant 80  “It [website] tells you step by step how you’re going to be treated which can help make the entire process less daunting” – Participant 68 | 8 | ✓ | ✓ |
|  |  |  |  | *Hindered Engagement* | | | | |
|  |  |  |  | Limited options to initiate care (e.g., only option is to make a phone call) | “I’m assuming I have to call and do it over the phone, which some people aren’t very comfortable with. Some people take a very long time to get help because they’re scared to talk to someone on the phone. So maybe having something where they can do it through their website can be helpful”- Participant 63 | 7 | ✓ | ✓ |
|  |  |  |  | Unclear or confusing steps to initiate care (e.g., differing steps for different services) | “I have no idea how to actually get services. Even though this is the services center, there’s nothing that really tells me how to make an appointment, as far as I’ve read. And maybe it could be hidden in one of these paragraphs but the fact that it’s not easily seen or accessible isn’t a good thing.” -Participant 61  “I don’t know if you call the number or email to set up an appointment, they don’t really make that super clear you have to dig for it”- Participant 45 | 14 | ✓ | ✓ |
|  | Website Goal |  | Statements related to the goal or purpose of the website that impacts engagement with the MH website. | *Promoted Engagement* | | | | |
|  |  |  |  | Engaging introductory page (e.g., diversity statement, patient centered, introduced services) | “It’s helpful that they list commitment to diversity it makes people feel more welcome to go there which is important”- Participant 45  “It summarizes a lot of information in a page, or like the contact information then what’s their purpose, and then I really like the ‘commitment to diversity.’”-Participant 85 | 20 | ✓ | ✓ |
|  |  |  |  | *Hindered Engagement* | | | | |
|  |  |  |  | Website was embedded in university website | “I get that it is part of a college so it’s a department, but I think it’s just confusing having ‘undergraduate’ or ‘graduate.’ It can turn people off that are looking for help. They might think it’s just a website for college students that are actually just taking classes.” -Participant 68  “I feel like this should be its own separate site. The admissions tab threw me off… I was just a bit confused… If I was clicking on the [university] Psychology Center, I’d want that to be a brand new page or tab.” -Participant 51 | 20 | ✓ | ✓ |
|  |  |  |  | Confusing introductory pages | “I guess I’m just a little bit confused because I’m like wait is this the only webpage about it right? Because the rest of it is school related?”- Participant 82 | 14 | ✓ | ✓ |
|  |  |  |  | Research-focused introductory pages | “If I was a regular person researching about what service is accessible to me when I click on this page I would immediately think that this is a research page or that it’s not a resource open to me- Participant 76  “It’s mostly research and if you wanted to get help, I think it’s a little harder. It’s less focused on getting actual help as much as research.” -Participant 52 | 6 | ✓ | ✓ |
| Promotion Strategies | Website Layout | Color | Statements related to the color schemes used on the website that impacts engagement with the MH website. | *Promoted Engagement* | | | | |
|  |  |  |  | Appealing color scheme in some website features (e.g., blue, yellow, green, red, and warm and pastel colors) | “Green is sort of the symbol for tranquility and calm so maybe add more details like that [green tabs]”- Participant 57  Calming colors like blue [pointed to blue tabs] or maybe even green are good.” -Participant 51 | 13 | ✓ | ✓ |
|  |  |  |  | *Hindered Engagement* | | | | |
|  |  |  |  | Unpleasant color scheme used throughout website (e.g., gray, black, dark blue, muted colors | This website is super plain, there’s really no color to it the basic colors are what just looks like navy blue, white, grey, and black so there’s really nothing happy about it, it’s just a depressing website”- Participant 25  “I don’t really like the red. Red’s a bit aggressive.” – Participant 54  “It needs a little bit more variety of color. I feel like everything’s somewhere along the grayscale.” -Participant 39 | 17 | ✓ | ✓ |
|  |  |  |  | Color of text blended with background | “The titles should have been a different color at least because it all kind of just blends in for me.” -Participant 61 | 4 |  | ✓ |
|  |  |  |  | Large amount of white space | “A huge white space is a waste of space.” -Participant 45  “I would probably improve it by making this section fill the entire page. I think it makes it look more professional and reliable as a source instead of having it [text] be a small area in the middle of the screen and everything else white.”- Participant 82 | 3 | ✓ | ✓ |
|  |  | Text and Font | Statements related to how the text and font of website visually impacts engagement with the MH website. | *Promoted Engagement* | | | | |
|  |  |  |  | Proper use of features that emphasize information (e.g., bolding, italics, larger font size, and underlining) | “This being in a bigger font and bolded… If it were super small and in the middle of the page, I might not see it and gloss over it and move on. I think it’s pretty important that they highlighted that there.” -Participant 51  “I do like that they do have in bold lettering program management assistant and then contact the clinic”- Participant 45 | 12 | ✓ | ✓ |
|  |  |  |  | Brief statements | “Those introductory paragraphs they’re short and divided into several parts I think that really helps me to follow what they’re talking about” – Participant 5  “In a short, succinct manner, they were able to get all their points across which I really appreciated. The content was there and communicated in an effective way.” -Participant 54 | 6 | ✓ | ✓ |
|  |  |  |  | *Hindered Engagement* | | | | |
|  |  |  |  | Large amounts of text | “It would be a lot nicer if there were a lot less words and there were just links to what was going on instead of a crap ton of words on here that are very hard to navigate”- Participant 25  “I just have to do a lot of reading. It’s so wordy and I think if I were seeking help, I would just not want to read all this.” -Participant 51 | 17 | ✓ | ✓ |
|  |  |  |  | Font was unappealing (e.g., size too small and type) | “The font is very small. I had to move my screen closer… it’s kind of tough to read.” -Participant 1  “Ok I guess here super small print you would think you would want to make that bigger” -Participant 45 | 8 | ✓ | ✓ |
|  |  |  |  | Poor use of features that emphasize information (e.g., bolding, italics, underlining, and asterisks) | “It’s really hard to understand what’s going on with the website because the bolding of the letters is just very confusing”- Participant 25  “To be bolding all these things is not really putting more emphasis on it. It’s just a lot of information that seems to be important but really is a lot to read.” -Participant 9 | 12 | ✓ | ✓ |
|  |  | Images and Visuals | Statements related to the images and visuals of the website that impacts engagement with the MH website. | *Promoted Engagement* | | | | |
|  |  |  |  | Included some relevant images (e.g., map, individual speaking to therapist, people smiling/looking happy, and logos) | “I really liked how it had such an easy way of seeing how to get to this place because I think that’s sometimes so confusing like where do I go and how do I get there.”- Participant 8  “I like that there’s a picture of what I’m assuming are actual people or students that go there, so I do think this is very welcoming. Even though this is a lofi photo, I still think that it’s welcoming to see humans.”-Participant 84  “I do like this icon or this logo”- Participant 2 | 20 | ✓ | ✓ |
|  |  |  |  | *Hindered Engagement* | | | | |
|  |  |  |  | Had unappealing (e.g., outdated and poor quality images) and irrelevant images (e.g., pictures of brain) | “I don’t like how blurry these photos are.” -Participant 53  “Why is there a picture of a building here? I don’t understand.”- Participant 55  “Digital art is usually better than random pictures of people sitting there. I see that and  I think that was probably staged because I don’t think you’re really allowed to take pictures of someone while they’re in therapy. I don’t really see the point in taking a picture of people sitting on a couch” -Participant 74  “This section should feature more human-centered photos or just photos with humans inside, because if I’m looking for mental health services and the first thing I see is a huge brain then I don’t know how to relate to that.” -Participant 73 | 22 | ✓ | ✓ |
|  |  |  |  | Lacked images (e.g., photos of staff) | “There’s no pictures. It looks too boring, I wasn’t really intrigued by anything”- Participant 42  “I don’t know what other content someone would want besides maybe seeing pictures of the therapist and info about the therapist. That’s something I didn’t really see when I was looking.” -Participant 62  “If it looks like some medical website with no images it’s not going to feel very welcoming but not overdone like I know sometimes the graphics can be overdone with the Gen Z push and it’s like you still want it to be legit”- Participant 45 | 22 | ✓ | ✓ |
|  |  | Organization | Statements related to the order, organization, and structure of information that impacts engagement with the MH website. | *Promoted Engagement* | | | | |
|  |  |  |  | Attempted to group information in sections (e.g., used clear heading titles or bullet points to organize information) | “I really liked this whole section, how it is appealing, its separated into different sections with treatments, consultations.”- Participant 7  “I liked how everything was put together in sections because if they’re all opened up, it would be a huge wall of text and that can be overwhelming for a lot of people. So, it was nice that it was put into these little blocks so you can take it one step at a time or if you’re looking for just one thing in particular, you can just go there directly.” -Participant 61 | 15 | ✓ | ✓ |
|  |  |  |  | Placement of important information (e.g., fees) is visible | “Since it is the home page, I definitely like how there’s already a ‘go to setting up an appointment with a mental health specialist or therapist’ so, no offense to the other website, I kind of like this one – I guess so far I like that the resource is already there, that you don’t necessarily look for it so I feel like that’s really good.”- Participant 2 | 14 | ✓ | ✓ |
|  |  |  |  | Attempted to ease navigation using tabs and headers | “The headers do a good job of summarizing what you’re looking for, so if you just want to browse through and only find the stuff you’re looking for, it’s very easy to do that.” -Participant 13  “The headings were nice. I think it was nice and it made the sections not feel too much and stressful to try to read. There is a good amount of words and if you’re trying to seek these resources, I doubt everyone reads the entire page, but breaking it up with these headings and them being a bigger size helps.” -Participant 35 | 22 | ✓ | ✓ |
|  |  |  |  | *Hindered Engagement* | | | | |
|  |  |  |  | Need for improved or additional navigation features (e.g., hyperlinks, dropdown menus, bars, and tabs/headers) | “I feel like a setup where if they had a bar on the side or the top that you could click to a specific section just so you know that on this page, you could find the hours and you can find this and that. Some people might just read this (long text) and be like ‘ok’ and not even keep looking.” -Participant 35  “Maybe some people would like it if it was broken up more, like if you could hide certain sections just so it can seem not as daunting. Even though this isn’t tons and tons of information, if you scroll like this really quickly, you might think it’s too much.” -Participant 55 | 29 | ✓ | ✓ |
|  |  |  |  | Information poorly placed and grouped (e.g., related information such as types of services not grouped together) | “This contact info should be higher and then hours” – Participant 30  “It’s all on one page, you’d have to dig for any of your information that you’re looking for, which is just not really helpful”- Participant 45  “If I was just going through stuff really fast, I might not even scroll all the way down to see services. I think it would be better if services offered was right here grouped and I could look at that immediately.” -Participant 85 | 32 | ✓ | ✓ |
|  |  | Interactive Components and Features | Statements related to interactive components or features that impacts engagement with the MH website. Interactive components refer to features that allow a two-way flow of information between a computer and a computer-user; responding to a user’s input. | *Promoted Engagement* | | | | |
|  |  |  |  | Included FAQ section | “The frequently asked questions tab is nice, too, because when something’s a frequently asked question that you have, it makes you feel better about having that question. It’s like ‘oh okay I’m not the only one.’ It also makes it very clear that these first-time things are something people usually get nervous about. It’s nice it walks through that in a very informal way.” – Participant 13  “This FAQ is cool. It’s very easy to understand and informational.”- Participant 4 | 10 | ✓ |  |
|  |  |  |  | Hyperlinked sections | “I love how they used the hyperlink because it’s just really straightforward to lead you. If this sentence has information that you’re interested in, then it’s great how they included a hyperlink in that sentence so you can go straight to it. Sometimes I’ll go through a website and if it’s a really long one, I’ll forget the parts that I thought were interesting. This is really helpful because I can just click it and it’ll open it in a separate window and just keep it there while I keep reading.” -Participant 12 | 2 | ✓ | ✓ |
|  |  |  |  | *Hindered Engagement* | | | | |
|  |  |  |  | Lacked search bar | “I wish it [website] had a search bar because it could be useful to search for other things”- Participant 61 | 2 |  | ✓ |
|  |  |  |  | Lacked other interactive features (e.g., filters and surveys) | “I would just make it a little bit more user-friendly in terms of maybe to have some kind of form, for example, here to ask about your psychological concern, your availability, what you’re looking for. That can be helpful because if a person is in emotional distress, they’re already most of the time having a really hard time focusing and having to fish out the information they need might be problematic for them. If there was a little more help, something to guide them in the right direction, that would be really nice.” -Participant 82  “This is not very interactive, I wasn’t sure if I wanted to read through all of this just to get some information”- Participant 45 | 5 | ✓ | ✓ |
|  | Language |  | Statements related to the language used in the websites that impacts engagement with the MH website. This code includes complicated language that leads users to search general web for clarification. | *Promoted Engagement* | | | | |
|  |  |  |  | Concise language | “I think it’s a concise website which is sometimes good because people just want to get to the point”- Participant 51 | 3 | ✓ | ✓ |
|  |  |  |  | *Hindered Engagement* | | | | |
|  |  |  |  | Used jargon | “They’ll say like dialectical behavior therapy or mindfulness-based stress reduction, but a lot of people maybe don’t know what that is and they don’t really explain it from what I saw”- Participant 49  “The title itself (Cognitive Behavioral Therapy Research and Training Clinic) is a little unfriendly for users. If I wasn’t a psych or cog sci student, I don’t know if I would understand.” -Participant 73 | 7 | ✓ | ✓ |
|  |  |  |  | Inaccessible to non-English speakers | My parents’ first language is Mandarin, so back to what I said about the content not being super beginner-friendly, having too much jargon, and it’d be too hard for them to read. I feel like there’s better options for my parents especially with the county- or city-sponsored websites. They probably have other languages or therapists that are able to speak in another language. As a customer or potential patient, that would be more appealing to me.”-Participant 73 | 9 | ✓ | ✓ |
|  | Important  Information | Testimonials | Statements related to testimonials included from previous clients, users, and service recipients impact engagement with the MH website. | *Promoted Engagement* | | | | |
|  |  |  |  | Included testimonials from previous clients | “I like that there’s some kind of testimony, like current people who have gotten treatment from them and what they’ve said, I think that’s really good”-Participant 5 | 4 | ✓ |  |
